# An Interesting Case of Inflammatory Myofibroblastic Tumor Masquerading as Lymphoma Detected on 18F-FDG PET-CT

**DOI:** 10.7759/cureus.44652

**Published:** 2023-09-04

**Authors:** Ritwik Wakankar

**Affiliations:** 1 Nuclear Medicine, Max Super Speciality Hospital, Delhi, IND

**Keywords:** fdg pet-ct, (18f)-fdg pet, inflammatory myofibroblastic tumor (imt), lymphoma, 18f-fluorodeoxyglucose positron emission tomography (18f-fdg pet)

## Abstract

Inflammatory myofibroblastic tumors (IMT) are a rare set of tumors that have been reported in various areas of the body but not quite as often in systemic lymph nodes. Herein, we discuss the case of a 60-year-old woman who presented with bilateral cervical and axillary lymphadenopathy and a low-grade fever. She subsequently underwent a fluorine-18-fluorodeoxyglucose positron emission tomography-computed tomography (18F-FDG PET-CT) as part of her evaluation. The scan revealed multiple hypermetabolic cervical, axillary, mediastinal, retroperitoneal, pelvic, and inguinal lymph nodes of various dimensions scattered throughout her body. Based on these findings, she was erroneously diagnosed to have lymphoma. It was only after histopathological correlation was the diagnosis revised to that of IMT, after which she was started on a course of oral corticosteroids. On follow-up imaging, she showed evidence of complete resolution of the involved lymph nodes. She has been disease-free for the past nine months after completing treatment. This case highlights the importance of including IMT as part of the differential diagnosis in suspected cases of lymphoma, giving credence to the phrase “all that glitters is not gold.”

## Introduction

Inflammatory myofibroblastic tumors (IMT) are mesenchymal tumors composed of neoplastic myofibroblasts and a conspicuous inflammatory infiltrate. These can arise in any part of the body (mostly in the lungs, liver, and gastrointestinal tract) and are mostly seen in children and young adults [1]. Throughout the literature, IMTs have been called by many names, such as inflammatory pseudotumor, plasma cell granuloma, pseudosarcoma, lymphoid hamartoma, and inflammatory myofibrohistiocytic proliferation, to name a few [2]. All of these names just go to show how little we actually know about the true nature of this disease. Few cases exist that have reported IMT with the exclusive involvement of systemic lymph nodes [3].

Conventional imaging modalities like ultrasonography (USG), computed tomography (CT), and magnetic resonance imaging (MRI) are commonly used while evaluating disease but are unable to differentiate between benign and malignant lesions, nor are they great at differentiating one malignancy from another, a fact especially highlighted when dealing with IMTs [4]. However, fluorine-18-fluorodeoxyglucose positron emission tomography-computed tomography (18F-FDG PET-CT) is being increasingly used in the imaging of malignancies such as lymphoma. A limited number of studies exist that have highlighted the utility of 18F-FDG PET-CT in IMTs [1,4-6], whereas it is even rarer to find literature regarding the role of 18F-FDG PET-CT in the assessment of primary IMT of the lymph nodes [7,8]. Here, a case of primary IMT of the systemic lymph nodes detected on 18F-FDG PET-CT is presented.

## Case presentation

**Figure 1:**
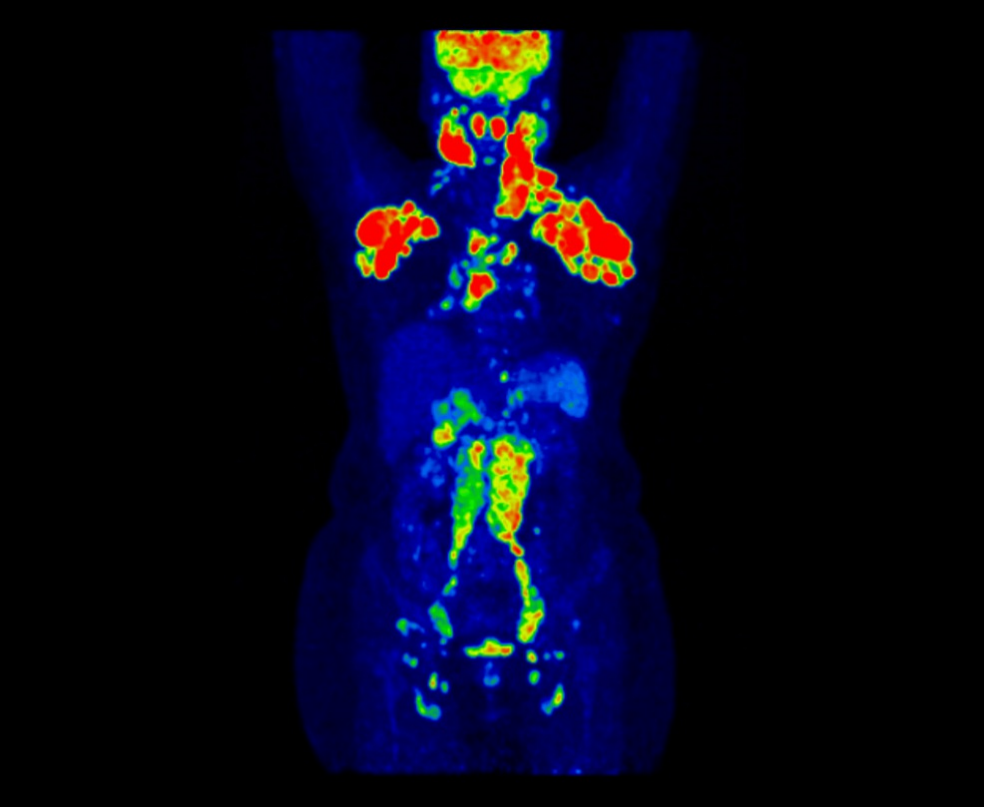
Maximum intensity projection (MIP) image showing multiple hypermetabolic lymph nodes on both sides of the diaphragm

A 60-year-old woman (height: 5’6” and weight: 67 kg) presented to the clinic with chief complaints of painless nodular swelling on both sides of her neck for the last three months with an associated low-grade fever. There was no history of being diagnosed with tuberculosis (TB) in the past or of coming in contact with anyone who had been diagnosed with TB. Her physical examination revealed multiple firm, nodular, mass-like lesions on both sides of her neck, her bilateral axillae and bilateral inguinal regions, which were thought to be enlarged lymph nodes. Her complete blood count (CBC), liver function test (LFT), and renal function test (RFT) were all within normal limits. An excisional biopsy was taken from one of the cervical lymph nodes on the right side of the neck. The histopathology report revealed the specimen to contain spindle-shaped myofibroblasts with a diffuse inflammatory infiltrate. Immunohistochemical (IHC) analysis of the specimen revealed the presence of smooth muscle actin (SMA) and Ki67 and an absence of anaplastic lymphoma kinase (ALK). Following this, an 18F-FDG PET-CT scan was done to assess the disease extent, revealing multiple hypermetabolic lymph nodes distributed throughout the body, as shown in the maximum intensity projection (MIP) image (Figure 1).

**Figure 2:**
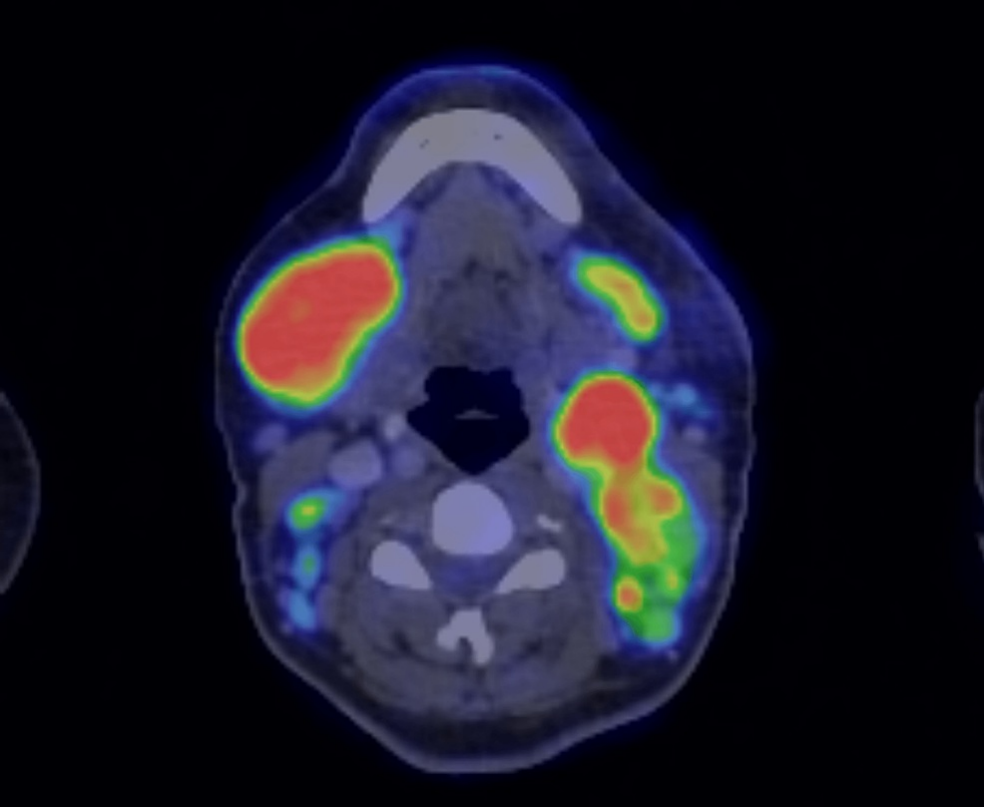
Multiple hypermetabolic bilateral enlarged cervical lymph nodes

**Figure 3:**
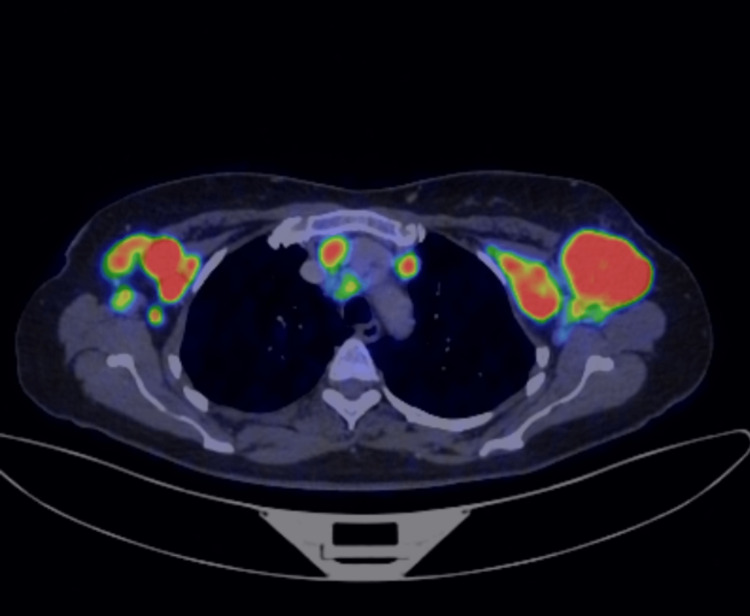
Multiple hypermetabolic enlarged bilateral axillary and mediastinal lymph nodes

**Figure 4:**
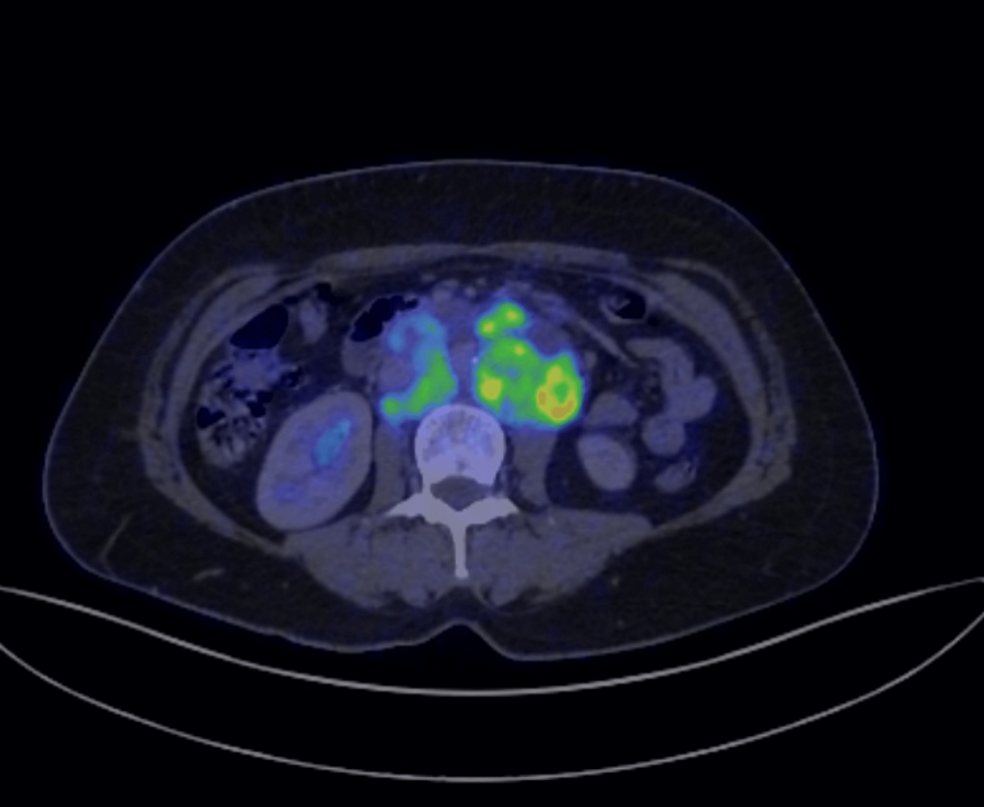
Multiple hypermetabolic and conglomerated retroperitoneal lymph nodes

**Figure 5:**
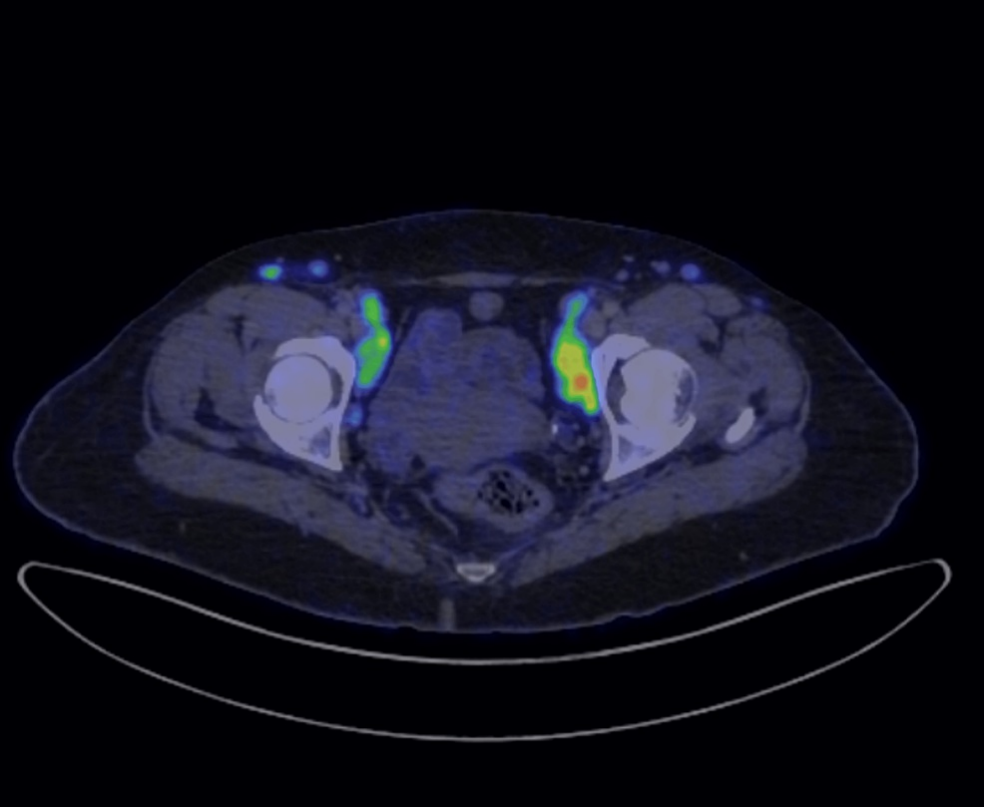
Multiple hypermetabolic bilateral pelvic and inguinal lymph nodes

In selected transaxial PET-CT images, multiple hypermetabolic cervical (SUVmax:12.1), axillary (SUVmax:10.1), mediastinal (SUVmax: 6.7), retroperitoneal (SUVmax: 5.5), pelvic (SUVmax: 6.0), and inguinal (SUVmax: 2.7) lymph nodes can be seen (Figures 2-5).

Based on her medical history and the 18F-FDG PET-CT findings, an initial diagnosis of lymphoma was made. However, this was revised once the histopathology report came out. Due to the extensive nature of the disease, a complete lymph node excision was not possible. She was started on daily oral corticosteroids (a dose of 0.5 mg/kg, prednisone) for one month, following which a follow-up scan revealed complete resolution of the disease. The patient has been disease-free for the last nine months since her last treatment.

## Discussion

According to the World Health Organization (WHO), IMTs are considered to be mesenchymal neoplasms of intermediate malignant potential. The etiologic factors responsible for the pathogenesis of IMTs are unclear. The radiological features of IMTs are also very varied and non-specific [9]. Many authors have reported cases of IMT exhibiting increased FDG uptake on PET-CT due to it being a metabolically active neoplasm [2,4,5]. IMT positivity on 18F-FDG PET-CT can be explained by the fact that the FDG is taken up by the tumor cells, macrophages, granulocytes, and inflamed tissues. Three factors have been shown to lead to an increased FDG uptake in IMTs, namely, high tumor cellularity, the presence of nuclear atypia, and a high proliferation index [10]. Gandhi et al have reported a case of IMT which presented as inguinal lymphadenopathy with fever. Initially, it was thought to be lymphoma but was revealed to be IMT on IHC [7]. Differentiating between lymphoma and IMT is difficult based solely on the clinical and imaging findings. The presence of SMA-positive myofibroblasts is essential for the differentiation from lymphoma, which is always SMA-negative. IMTs are also known to show several mutations in genes encoding anaplastic lymphoma kinase (ALK), receptor tyrosine kinase ROS1, and platelet-derived growth factor receptor beta (PDGFRβ) to name a few. ALK gene mutations are especially important, as they are seen in 50% of IMTs and can serve as targets for immunotherapy using crizotinib in IMT patients who fail to respond to conventional treatments [11,12]. It is difficult to differentiate between lymphoma and IMT as is demonstrated in this case, however, certain clinical findings can help guide us in the right direction. Lymphoma has a bimodal age distribution, with peaks in young adulthood (20-40 years) and old age (60 years and above), whereas IMTs are more common in young adults and children, although they can occur at any age. Lymphomas present with a more aggressive course with fever, night sweats, and weight loss while IMTs can present with organ-specific symptoms like respiratory distress, bowel obstruction, etc. On imaging, using computed tomography (CT), magnetic resonance imaging (MRI), and 18F-FDG PET-CT, lymphomas present with multiple enlarged lymph nodes, hepatosplenomegaly, and bone marrow lesions while IMTs tend to show less widespread lymph nodal involvement. However, as has been described in this case report and in a few others as mentioned earlier, this is not always the case. A definite distinction between lymphoma and IMT can only be made based on histopathology and IHC, where lymphomas stain positively for CD20 and CD30, whereas IMTs stain positively for SMA, ALK, ROS1, etc.

The treatment options for IMT include surgery, radiation therapy, chemotherapy, and steroids. Among these, complete surgical resection is the most optimal treatment option. However, that could not be done in this patient due to the extensive nature of her disease. She was, therefore, started on daily oral prednisone for a duration of one month, following which she went into complete remission and has been disease-free ever since.

## Conclusions

Systemic IMT of the lymph nodes is a rare disease that can prove to be a diagnostic challenge at times. They mimic lymphoma, as both are very FDG-avid. This case report aims to show that IMT must be included in the differential diagnosis of patients suspected of having lymphoma. It also shows that oral corticosteroid therapy can have some role to play in its treatment.
